# Analysis of cerebral glucose metabolism following experimental subarachnoid hemorrhage over 7 days

**DOI:** 10.1038/s41598-022-26183-1

**Published:** 2023-01-09

**Authors:** Fabian Schadt, Ina Israel, Alexandra Beez, Kastriot Alushi, Judith Weiland, Ralf-Ingo Ernestus, Thomas Westermaier, Samuel Samnick, Nadine Lilla

**Affiliations:** 1 Department of Nuclear Medicine, University Hospital Würzburg, Oberdürrbacher Str. 6, 97080 Würzburg, Germany; 2 Department of Neurosurgery, University Hospital Würzburg, Josef-Schneider Str. 11, 97080 Würzburg, Germany; 3 Department of Neurosurgery, University Hospital Magdeburg, University of Magdeburg, Leipziger Str. 44, 39120 Magdeburg, Germany; 4 Department of Vascular Medicine, German Aortic Center Hamburg, University Heart and Vascular Center, Hamburg, Germany; 5 Department of Neurosurgery, Helios-Amper Klinikum Dachau, Krankenhausstr. 15, 85221 Dachau, Germany

**Keywords:** Neuroscience, Diseases of the nervous system, Neurological disorders, Cerebrovascular disorders, Diagnosis, Positron-emission tomography

## Abstract

Little is known about changes in brain metabolism following SAH, possibly leading towards secondary brain damage. Despite sustained progress in the last decade, analysis of in vivo acquired data still remains challenging. The present interdisciplinary study uses a semi-automated data analysis tool analyzing imaging data independently from the administrated radiotracer. The uptake of 2-[^18^F]Fluoro-2-deoxy-glucose ([^18^F]FDG) was evaluated in different brain regions in 14 male Sprague–Dawley rats, randomized into two groups: (1) SAH induced by the endovascular filament model and (2) sham operated controls. Serial [^18^F]FDG-PET measurements were carried out. Quantitative image analysis was performed by uptake ratio using a self-developed MRI-template based data analysis tool. SAH animals showed significantly higher [^18^F]FDG accumulation in gray matter, neocortex and olfactory system as compared to animals of the sham group, while white matter and basal forebrain region showed significant reduced tracer accumulation in SAH animals. All significant metabolic changes were visualized from 3 h, over 24 h (day 1), day 4 and day 7 following SAH/sham operation. This [^18^F]FDG-PET study provides important insights into glucose metabolism alterations following SAH—for the first time in different brain regions and up to day 7 during course of disease.

## Introduction

Despite new discoveries in research and sustained advances in the treatment of aneurysmal subarachnoid hemorrhage (aSAH), morbidity and mortality remain high^1,2^. The world-wide incidence of aSAH is approximately 7.9 per 100,000 person-years with aSAH accounting for about 5% of all strokes^3,4^. Overall mortality rate is estimated to be approximately around 40%^4,5^. Brain injury following aSAH is multimodal and occurs directly as early brain injury, and secondarily as delayed brain injury^6^. During the first 2 weeks following aSAH, angiographic cerebral vasospasm (CVS) occurs in about 70% of SAH patients, but only 30% of patients develop delayed cerebral ischemia (DCI). Therefore, DCI remain the major cause of morbidity and mortality among patients who survived the initial bleeding and treatment of the ruptured aneurysm^7,8^. Several mechanisms during the acute phase of SAH contribute to DCI and poor outcome. These include neuroinflammation, microthrombosis, cortical spreading depolarizations, disrupted blood–brain barrier (BBB) integrity, microvascular dysfunction and metabolic derangement^9–12^. Changes in brain metabolism and accumulation of metabolites such as lactate, pyruvate and glutamate are described, possibly responsible for a derangement of oxidative brain metabolism, leading to secondary brain injury and poor outcome^2,10,12^. Glucose is the main energy substrate of the brain and crucial for normal brain function. However, the effects of SAH on cerebral glucose metabolism are not fully understood yet. In this study we aimed to analyze how glucose metabolism in a well-established SAH rat model is affected by SAH in vivo using [^18^F]FDG-PET. Thereby, data evaluation and analysis was performed using our self-developed, semi-automated MRI-template based data analysis tool, as previously described in Schadt et al*.*^13^. This interdisciplinary study provides data on metabolic processing of glucose in the early phase following SAH, where so far only limited data is available, and for the first time measurements of [^18^F]FDG brain metabolism over course of disease up to day 7 following SAH and as well for the first time in separation of different brain regions. These gained insights on brain glucose metabolism should help to develop new targets for translational neuroprotective therapies following aSAH.

## Material and methods

### Animals and ethical statements

PET data of 14 male Sprague–Dawley rats (Harlan Winkelmann GmbH, Borchen, Germany) weighing 250–300 g (mean age 8 weeks) were analyzed. The animals were maintained for at least one week for acclimatization before induction of SAH, including a 12/12-h light/dark cycle. Access to food and water was given all time, except that at least six hours before PET measurement, food was removed. Induction of SAH was performed via the endovascular filament model as described previously^12,14^. In summary, a right paramedian longitudinal incision on the ventral neck was performed. The external carotid artery was ligated and exiting branches were coagulated. Afterwards, a temporary aneurysm clip was placed on the common and internal carotid arteries. The external carotid artery was incised about 6 mm distal from the carotid bifurcation and a 3.0 Prolene filament (Ethicon, Inc. Somerville, New Jersey) was inserted and secured with a silk ligature. Next, temporary clips were removed and the external carotid artery was cut. Thus, the filament could be moved intracranial via the internal carotid artery. The filament was then advanced 3 mm further perforating the vessel in the area of the anterior cerebral artery (ACA). Immediately, the filament was quickly pulled back into the external carotid artery ascertaining reperfusion of the internal carotid artery.

Induction of anesthesia was performed with 4% isoflurane, followed by oral intubation and mechanical ventilation with an air-oxygen mixture to provide normal blood gases. After induction of anesthesia, isoflurane was lowered to 2.5% for surgical procedures and to 1.5% from 30 min before induction of SAH until 30 min after induction of SAH. Thereafter, animals were woken up, returned to separate boxes for recovery and only got isoflurane anesthesia during [^18^F]FDG-PET scans (4% isoflurane for induction and 1- 2% isoflurane for maintenance, in oxygen at 2 L/min). Sham animals in the control group were treated and operated in the same way with a short temporal occlusion of the vessel and just quitting the perforation process of the ACA. Notably, the length and depth of anesthesia was performed in the same manner. In awareness that glucose metabolism has a circadian rhythm, the experiments were performed in precisely the same time frame of the day, starting 7 am in the morning, with 3 respectively 4 animals per day. In addition, the follow up [^18^F]FDG-PETs were performed in the same manner and order at the same time of the day. Temperature was constantly measured throughout the whole experiment, keeping the temperature level at 37 °C. Continuous arterial blood pressure measurement was performed by cannulating the tail artery. SAH was verified via right frontal ICP probe (data not shown). None of the operated animals had to be excluded nor was there an animal loss in this experimental series. The division of the animals into group (1) subarachnoid hemorrhage (SAH, n = 7) and (2) sham operated controls (sham, n = 7) was obtained by drawing lots. All experiments have been approved by the district government of Lower Franconia (Regierung von Unterfranken AZ: 55.2–2531.01–40/12) and have been carried out in accordance with directive 2010/63/EU and in compliance with the German animal protection law. Study results are reported in accordance with the ARRIVE guidelines.

### Preparation of 2-[^18^F]fluoro-2-deoxyglucose for analysis

2-[^18^F]fluoro-2-deoxyglucose ([^18^F]FDG) used in the present investigation was produced in-house at the Interdisciplinary PET-Centre (IPZ) of the University Hospital Würzburg using the GE-PETtrace cyclotron and the GE-Fastlab® synthesis unit (GE Medical Systems, Uppsala, Sweden) as previously described^15^. [^18^F]FDG was dissolved in physiological saline and analyzed for radiochemical purity by HPLC and TLC before application.

### Serial PET measurements

[^18^F]FDG-PET measurements were performed on a Siemens Inveon µPET scanner (Siemens Medical Solutions, Knoxville, USA) at 3 h, 1 day, 4 days, and 7 days after SAH induction and sham operation, respectively. Over the entire PET acquisition, animals were under anesthesia with 2% isoflurane in 100% oxygen. To avoid hypothermia, animals were warmed with a custom-made heating pad during the anesthetic period. The injected [^18^F]FDG had an activity of 31.4 ± 3.4 MBq and was injected intravenously into the anesthetized animal via the tail vein. Emission scanning time was set for 20 min, 40 min after [^18^F]FDG injection.

Data reconstruction was performed using default settings including OSEM2D algorithm (OSEM2D: Sinogram Rebinning Algorithm = Fourier Rebin*, Projection Filter = Ramp*, Projection Cutoff (Nyquist) = 0.5, Iterations = 4, EM Iterations = 0, Number of OSEM2D subsets = 16, Number of OSEM2D iterations = 4) provided by the embedded software package Inveon Acquisition Workplace (Version 1.5.0.28). After the emission scan, a transmission scan was also performed for attenuation correction. For data analysis purposes, the emission scan was divided in two time frames (10 min each). However, in the result section, only the time frame between 50 and 60 min is presented, since no significant differences between the two frames were apparent.

### Software programs

Reconstructed data was analyzed on a self-developed semi-automated nuclear medicine data processing analysis tool (NU_DPA) implemented in the software program Matlab (version 2018a, MathWorks, United States)^13^. In addition to pre-implemented functions in Matlab, the following functions, datasets as well as toolboxes were used for the evaluation of our data: Sprague Dawley T2*-weighted and atlas MRI data sets provided by *Papp *et al*.*, *Sergejeva *et al*.* and *Kjonigsen *et al*.*^16–18^ for anatomical sub-classification of the PET data, a tool for NIfTI data import by Shen et al*.*^19^, the Medical Image Registration Toolbox (MIRT) by Myronenko^20^ for affine registration processes, the VolumeViewer3D of the Medical Image Reader and Viewer Toolbox by *Schaefferkoetter*^21^ for data visualization and Export_fig by Altman^22^ for exporting high-resolution graphics. For the purpose of simplicity, this manuscript refers to the MR image dataset of *Papp *et al. as a T2*-weighted dataset, even though it is actually based on high-resolution, contrast-enhanced structural (including T2 and T2*-weighted) and diffusion-weighted MR images^17^.

### NU_DPA tool

As previously described in Schadt et al. (2021)^13^, the NU_DPA tool is a semi-automated MRI template-based data analysis tool. After some initial input (e.g. names of image datasets), the tool is designed to align all image datasets of an experimental study in a uniform procedure and to evaluate them taking also anatomical information into account. Thereby, all processing steps (e.g. registration processes) implemented in the tool are based on the MR image/atlas dataset combination added by the user.

In a first step of the data analysis, the high-resolution MR image datasets are adapted to the lower resolution of the PET image data by linear interpolation using the internal Matlab algorithm *imresize3.m*. In this way, the processing time of the data analysis, including the required working memory, can be greatly reduced. The resized MR image datasets then form the basis for the automatic segmentation of the volume-of-interest (VOI).

In this study, after segmentation of all VOI, a PET template was created from the experimental control group image datasets (sham), to which all PET image datasets were aligned using the MIRT algorithm. The PET template was then two times co-registered with the MR image dataset similar to^23^. Finally, all data already aligned to the PET template were then aligned to the MR image/atlas dataset combination using the transformation matrix created by the co-registration process.

### Statistical data analysis

Statistical evaluation was executed in NU_DPA using the semi-quantitative parameter uptake ratio (UR), according to the data analysis methods by Miederer et al*.*^24^. The UR was calculated from the ratio of the measured radioactivity in a subregion of the VOI (VOI_target_) to the measured radioactivity of the total VOI (VOI_wholebrain_).

$$UR= \frac{measured\, radioactivity\, in\, {VOI}_{target}}{measured\, radioactivity\, in\, {VOI}_{wholebrain}}$$

Due to the small number of animals (n = 7), no normal distribution was assumed and data was statistically evaluated using the non-parametric Mann–Whitney U-test. Bonferroni corrections were made for serial measurements and confirmed significance levels. P-values ≤ 0.05 were considered statistically significant.

## Results

According to *Schadt *et al*.*^13^, the PET image datasets were aligned to the resized T2*-weigthed as well as MR atlas image datasets which were adjusted to match the spatial resolution of the PET image datasets. Therefore, the MRI datasets were resized by a scale factor of 1/20 to match the resolution of the PET image datasets (25 mm × 25 mm × 25 mm versus 776 mm × 776 mm × 796 mm). Due to the poorer spatial resolution, the analysis of the PET image data is reduced to the five largest atlas regions of the MRI atlases of *Papp *et al., the gray and white matter, the neocortex, the basal forebrain region and the olfactory system.

**Figure 1:**
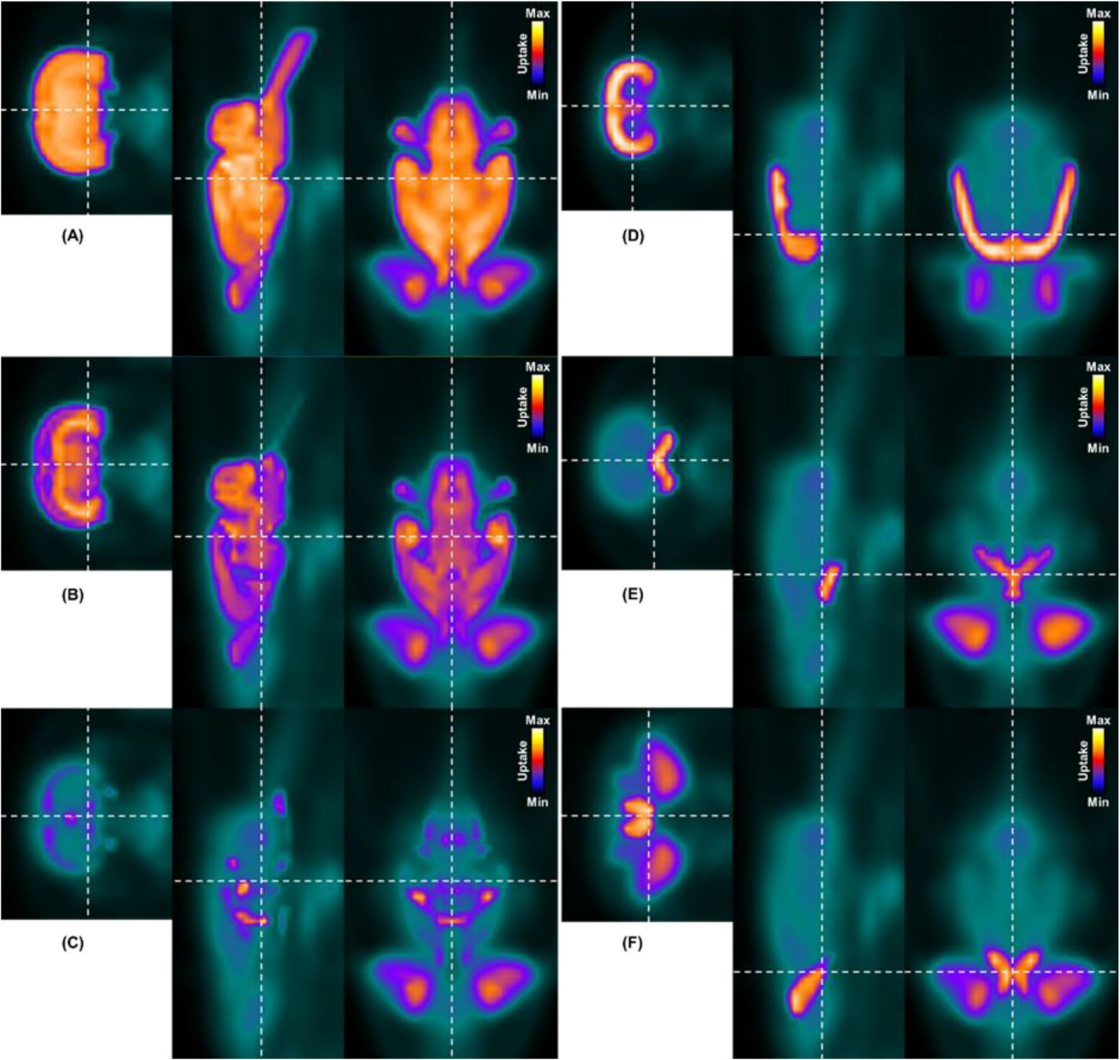
Co-registered PET image template based on the sham-operated controls as well as following anatomical image information: MRI T2* image (**A**), gray matter (**B**), white matter (**C**), the neocortex (**D**), the basal forebrain region (**E**) as well as the olfactory system (**F**).

Figure 1A shows a merged image of the PET template (consisting of the image datasets of sham-operated controls) and the resized T2*-weighted MR image of the whole brain after co-registration as a single-color display. The intensity values of both images (MR and PET) were adjusted for display in the merged image to highlight the respective MR regions in the PET template. In this context, Fig. 1B–F show the merged images of the PET template and one of each of the five largest MR atlas regions.

For display purposes, the images were again enlarged by the reciprocal scaling factor of 20 and highlighted in color.

**Figure 2:**
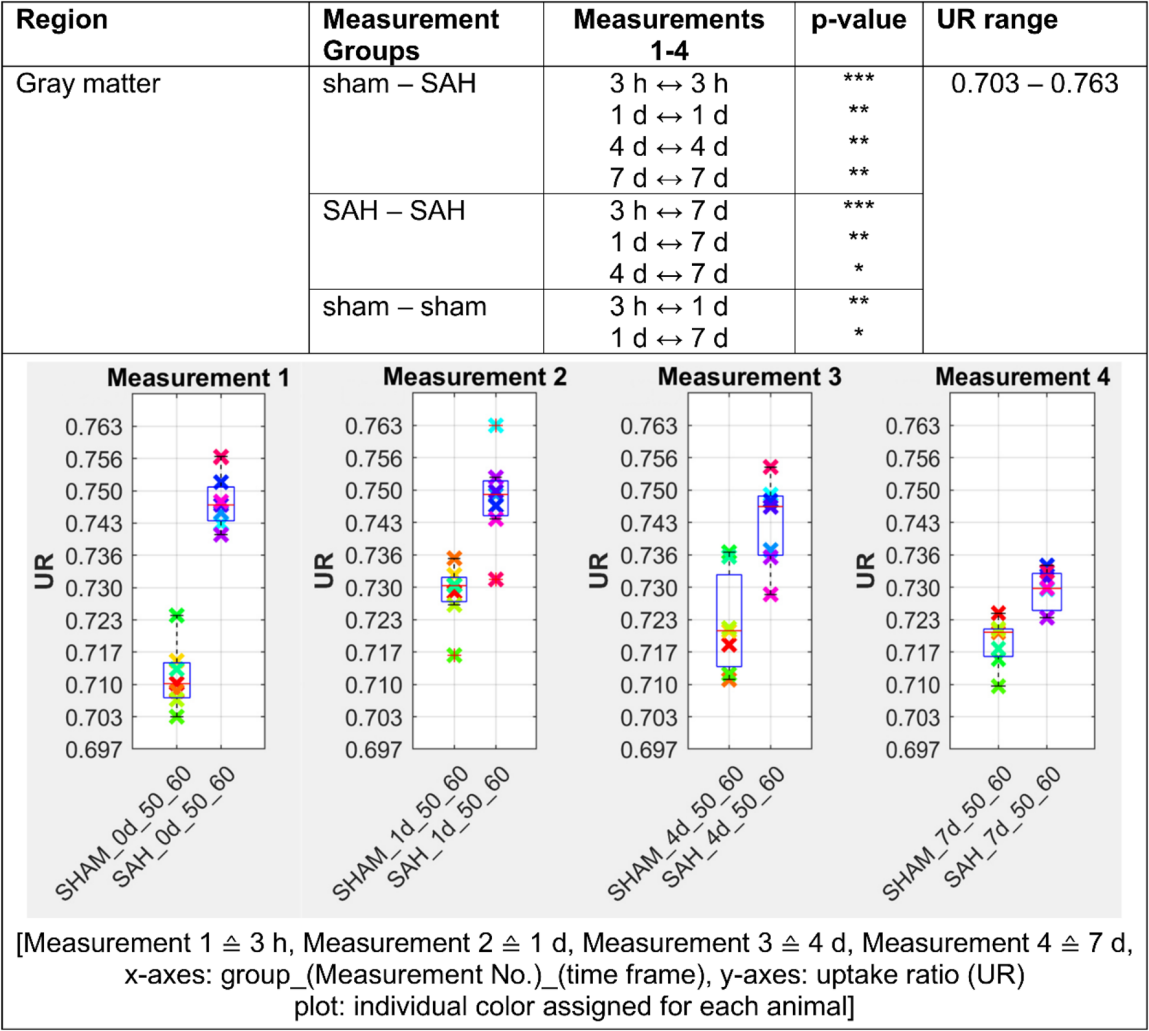
SAH animals show a highly significant increased [^18^F]FDG uptake ratio (p ≤ 0.001) compared to sham operated controls in the gray matter 3 h after SAH/sham operation. This effect persisted in the follow-up measurements and remained significantly increased (p ≤ 0.01) until day 7 after SAH/sham operation. p ≤ 0.05 *; p ≤ 0.01 **; p ≤ 0.001 ***.

**Figure 3:**
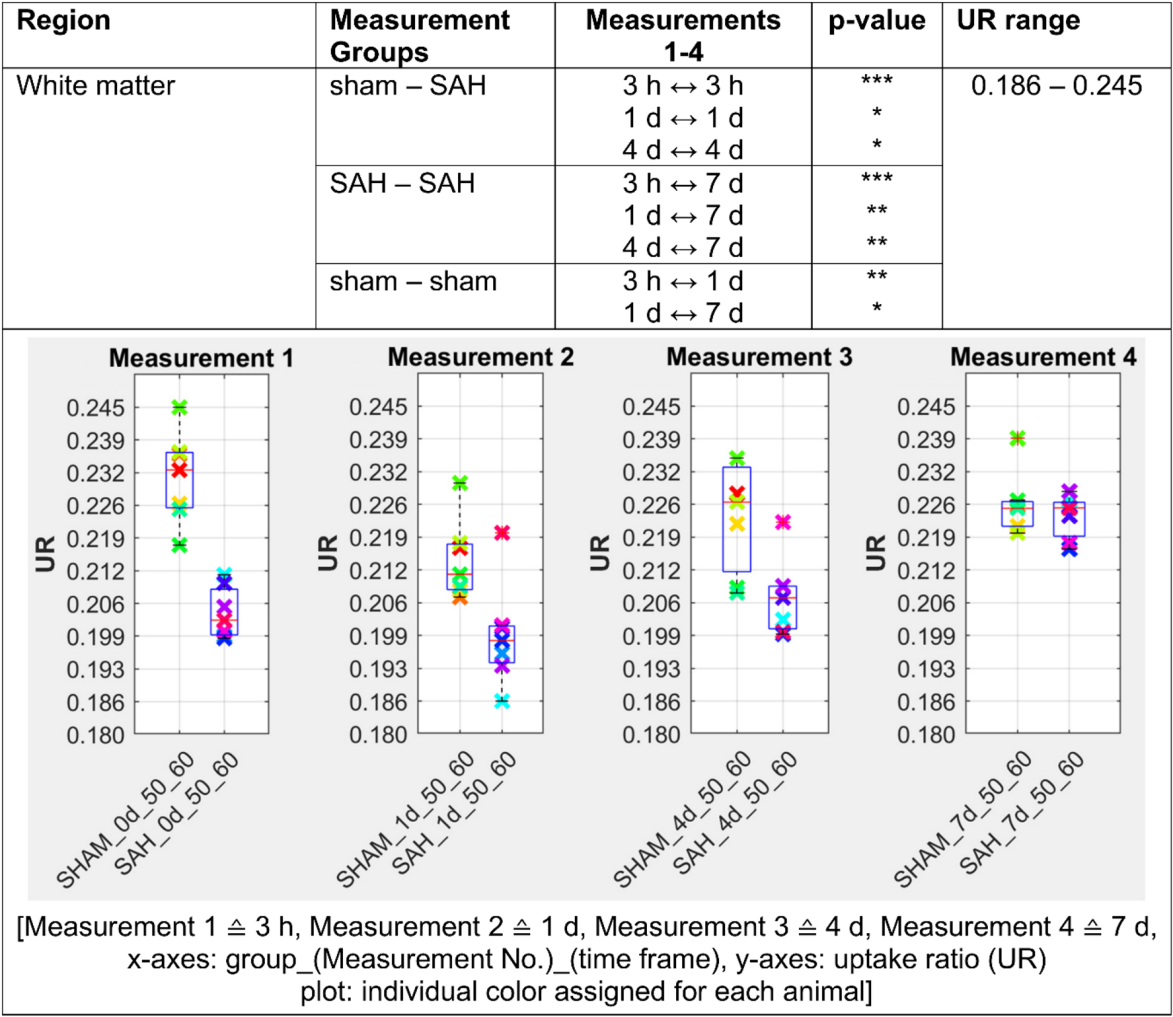
[^18^F]FDG uptake ratio is highly significant (p ≤ 0.001) reduced in white matter in SAH animals compared to the sham operated group 3 h following the SAH/sham operation. The UR of SAH animals remained significantly (p ≤ 0.05) reduced in white matter up to day 4 following SAH but converges strongly on day 7. p ≤ 0.05 *; p ≤ 0.01 **; p ≤ 0.001 ***.

**Figure 4:**
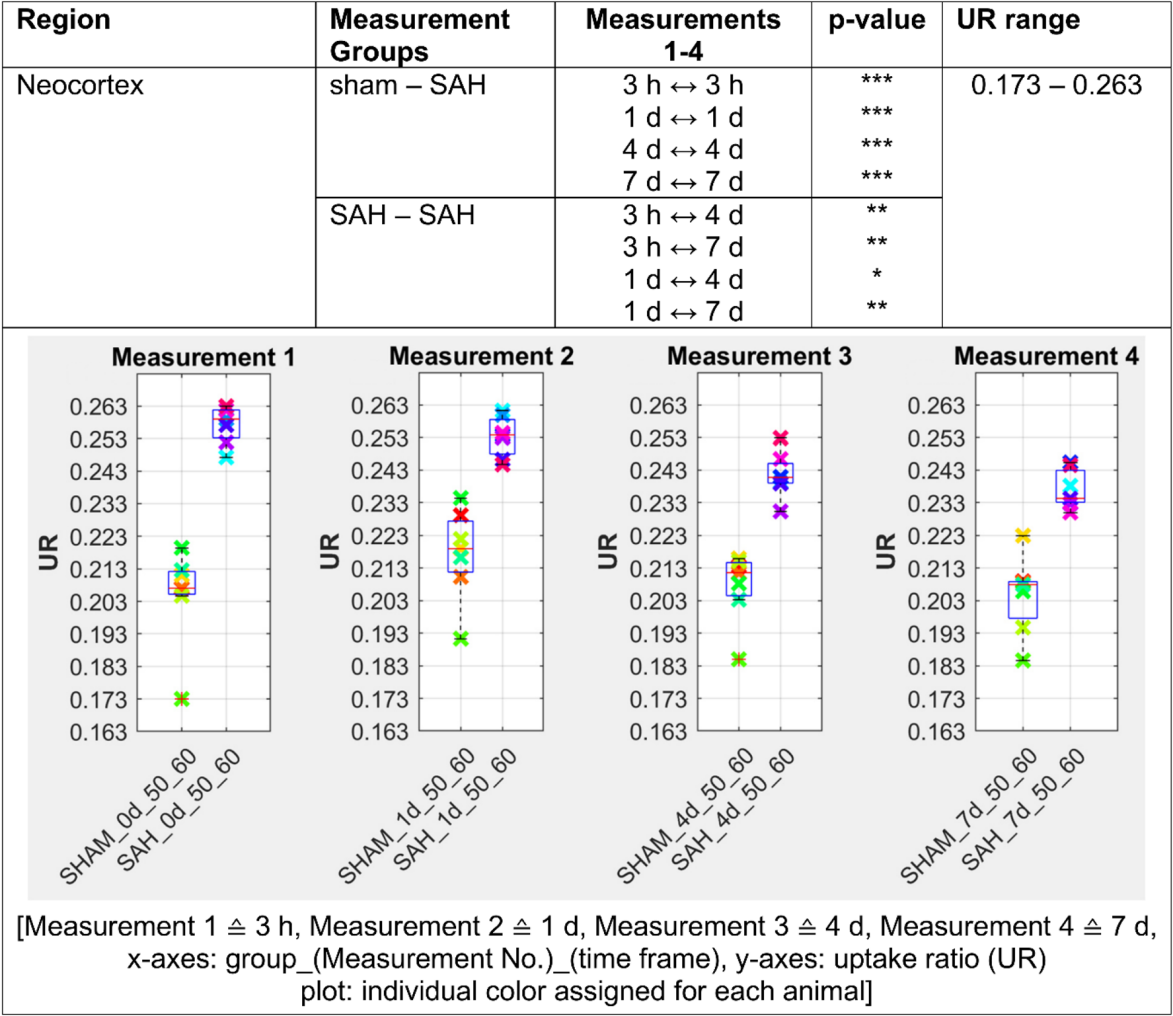
The [^18^F]FDG uptake in SAH animals is highly significant (p ≤ 0.001) elevated in the neocortex compared to the sham operated control and stays significantly higher (p ≤ 0.001) up to day 7 following SAH. p ≤ 0.05 *; p ≤ 0.01 **; p ≤ 0.001 ***.

**Figure 5:**
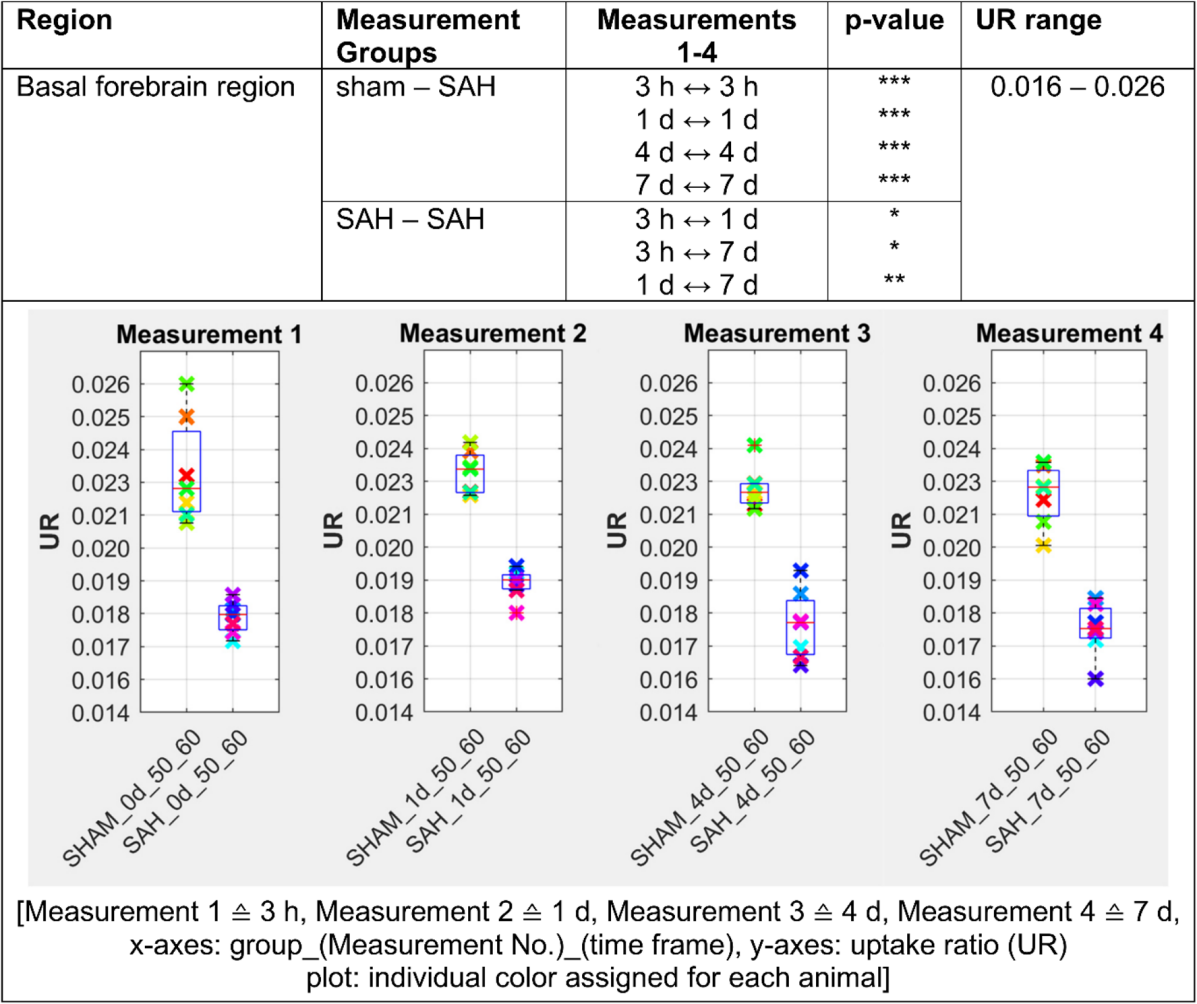
In SAH animals the [^18^F]FDG uptake ratio in the basal forebrain region is highly significant decreased (p ≤ 0.001) compared to sham operated controls throughout the 7-day study period. p ≤ 0.05 *; p ≤ 0.01 **; p ≤ 0.001 ***.

**Figure 6:**
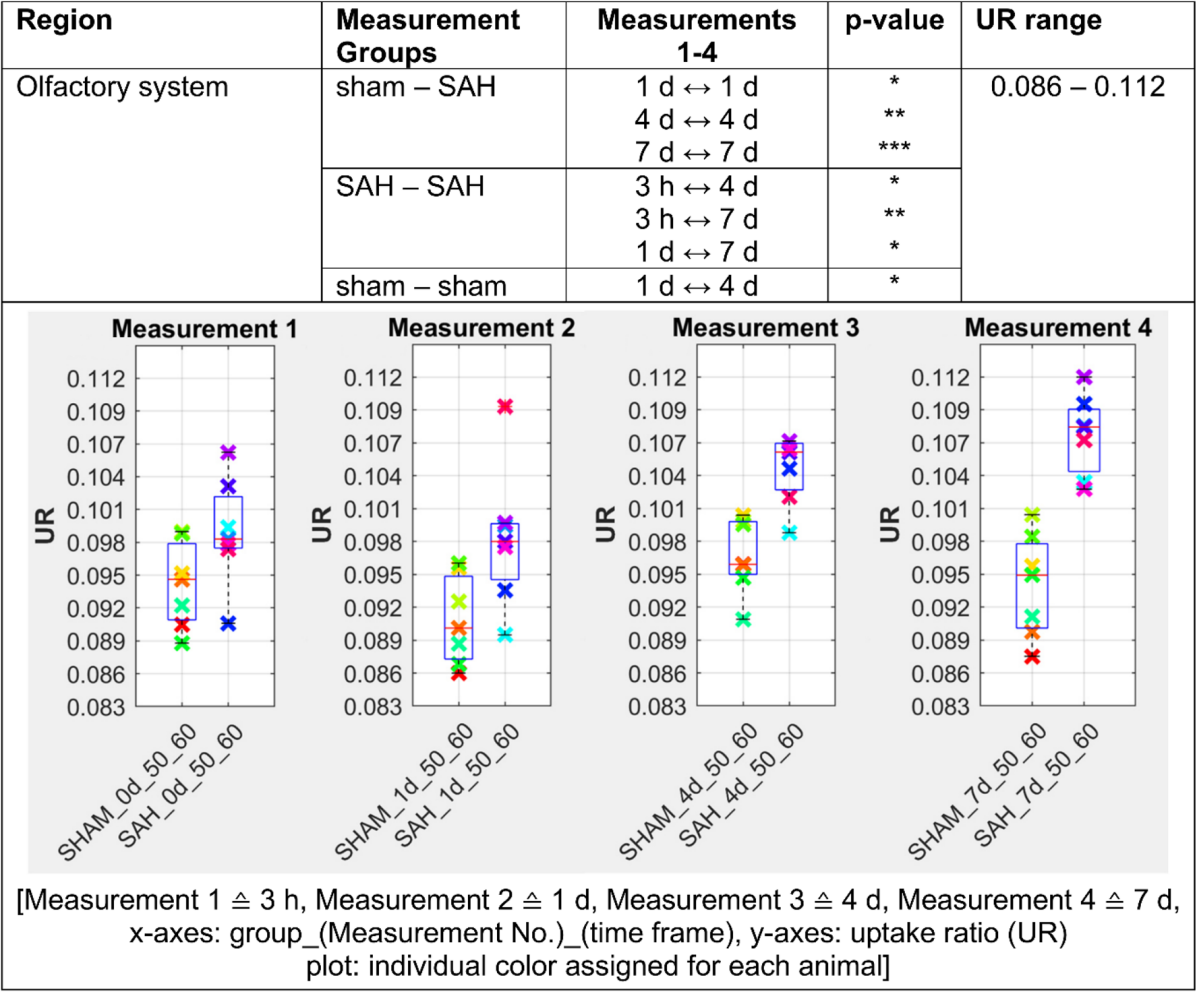
[^18^F]FDG uptake ratio is significantly elevated in olfactory system on day 1 following SAH compared to sham operated controls. This more-uptake gets even more significant on day 4 (p ≤ 0.01) and on day 7 (p ≤ 0.001) following SAH/sham operation. p ≤ 0.05 *; p ≤ 0.01 **; p ≤ 0.001 ***.

Figures 2, 3, 4, 5 and 6 presents the significant differences for gray matter, white matter, neocortex, basal forebrain region as well as the olfactory system. In the column *Measurement Groups*, the experimental groups, which were being compared, are listed. In the following two columns the individually measurement time comparisons as well as its significant p-values found are shown. Thereby, *Measurements 1–4* equals the time after SAH induction or sham operation. The last column (UR range) gives information about the value distribution of the [^18^F]FDG uptake ratio. The graphs below represent the individual UR value of each animal for each measurement. Each animal is assigned its own color by the NU_DPA tool and the individual values are shown for the respective time frames.

In our analysis, PET data showed a significant increase of [^18^F]FDG uptake in gray matter, neocortex and olfactory system of SAH animals compared to the sham operated group (p < 0.001 for 3 h and p < 0.01 for days 1, 4 and 7). Regarding white matter and basal forebrain we found a significant lower uptake of [^18^F]FDG in SAH animals compared to the sham operated group (p < 0.001 for 3 h, p < 0.001 for days 1–7 for the basal forebrain region and p < 0.01 for days 1–4 for the white matter).

**Figure 7:**
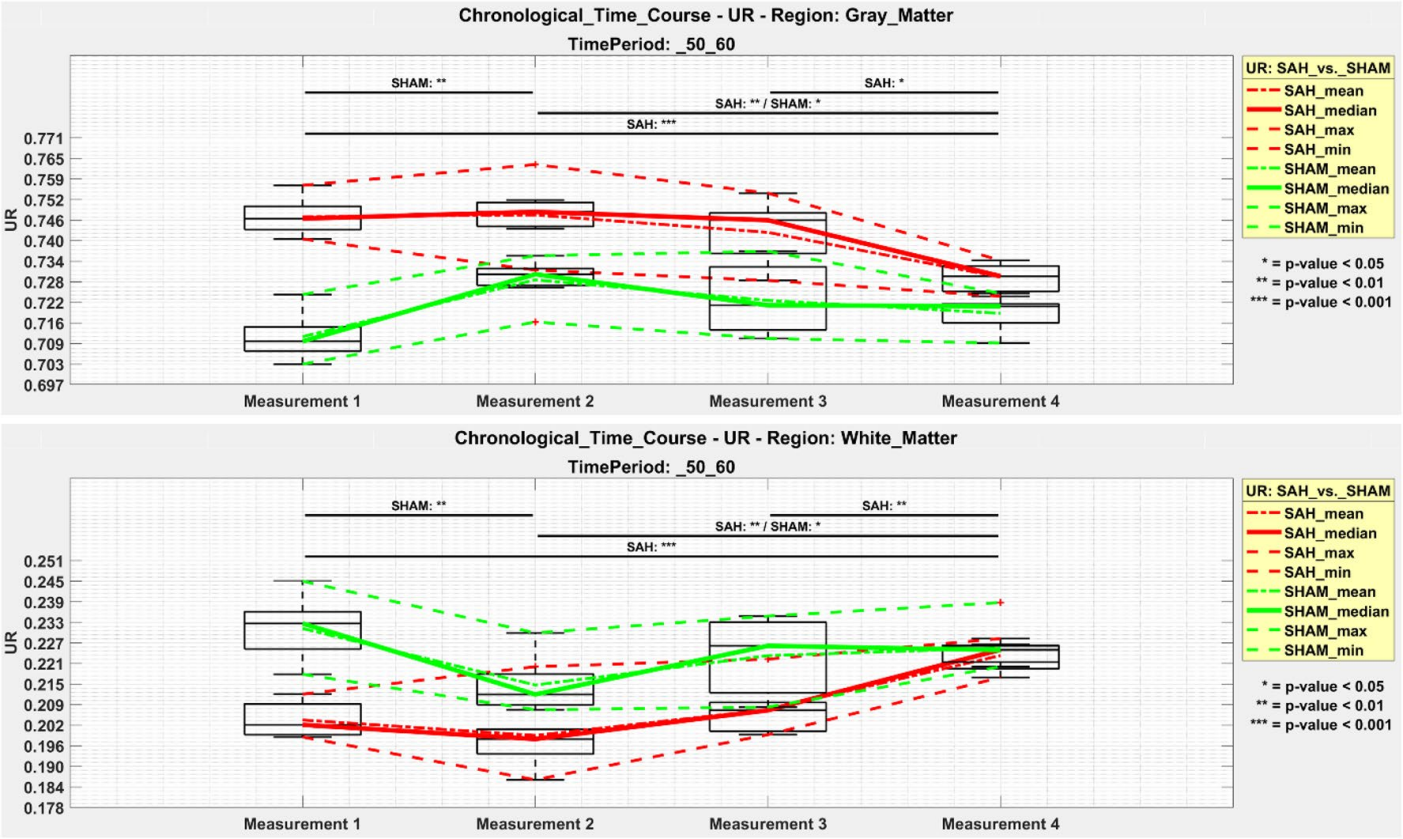
Chronological time course of [^18^F]FDG uptake in SAH animals (red lines) and sham operated controls (green lines) over course of disease/observation period for gray (top) and white matter (bottom). Measurement 1 = 3 h following SAH/sham operation; Measurement 2 = 24 h respectively day 1 following SAH/sham operation; Measurement 3 = day 4 following SAH/sham operation; Measurement 4 = day 7 following SAH/sham operation. UR = uptake ratio. p ≤ 0.05 *; p ≤ 0.01 **; p ≤ 0.001 ***.

With focus on the two largest regions, the individual UR value of each animal for each measurement (Figs. 2, 3) were additionally summed as chronological time course in one graphic automatically created by the NU_DPA tool (Fig. 7).

### Observed effects between the experimental SAH and sham group

SAH animals showed a highly significant increased uptake ratio of [^18^F]FDG (p ≤ 0.001) in gray matter compared to sham operated controls starting 3 h following SAH/sham operation and lasting significantly elevated (p ≤ 0.01) up to day 7 following SAH/sham operation. In white matter, SAH animals showed a highly significant (p ≤ 0.001) reduced uptake of [^18^F]FDG compared to sham operated controls already 3 h following SAH/sham operation, which remained significantly reduced (p ≤ 0.05) up to day 4 following SAH/sham operation.

### Observed time-related effects within each experimental group

Several significant effects on [^18^F]FDG uptake in gray matter were observed in the animals with SAH. Compared to [^18^F]FDG uptake ratio at day 7, [^18^F]FDG uptake ratio in gray matter was significantly increased 3 h following SAH (p ≤ 0.001). In the follow-up measurements, the observed effect decreased continuously in our SAH group, but stayed significant. For white matter, [^18^F]FDG uptake was significantly lower in the SAH group compared to the [^18^F]FDG uptake on day 7 (p ≤ 0.001). This observed effect decreased slightly in subsequent measurements, but still showed significant differences in [^18^F]FDG uptake between day 1 and day 7, and day 4 and day 7 in our SAH cohort. Our sham operated controls showed a significant lower [^18^F]FDG uptake (p ≤ 0.01) within the gray matter between 3 h and day 1 following sham operation as well as a significant higher radiotracer enrichment on day 1 compared to day 7. In white matter, these effects were opposite. After surgery, our sham operated controls showed significantly higher radiotracer accumulation in white matter 3 h after sham operation compared to day 1 post-op as well as a significantly lower [^18^F]FDG uptake at day 1 compared with day 7 (Fig. 7).

## Discussion

Our serial [^18^F]FDG-PET analysis shows a significant higher uptake of [^18^F]FDG in gray matter, neocortex and olfactory system of SAH animals compared to sham operated animals. This is in line with the only other published study on [^18^F]FDG-PET analysis in an experimental SAH rat model by *Song *et al*.*^25^, in which significant higher standardized uptake values (SUV) (p < 0.05) were determined in [^18^F]FDG-PET at 3 and 6 h after induction of experimental SAH compared to a sham group, indicating an increase in anaerobic glycolysis and a global hyper-glycolysis to balance the mismatch of energy need and supply, already in the first hours following SAH.

In a primary analysis (using the free software tool AMIDE), we also evaluated imaging data by calculating the standardized uptake value (SUV), whereby similarly to Song et al*.* only significant effects were observed between the (1) experimental SAH and (2) sham operated groups 3 h following experimental SAH, and only a trend in elevated [^18^F]FDG uptake in the SAH group on day 1, day 4 and day 7 could be observed (data not shown). However, when comparing the ratio of measured radioactivity using the uptake ratio, we could observe further significant differences between both experimental groups and in different brain regions.

We assume that the strong differences in the statistical data analysis using SUV compared to UR are to be attributed to two main aspects: first, SAH is a disease affecting the entire metabolism in the brain. Therefore, the tracer accumulation of [^18^F]FDG might be (severely) impaired. Second, the SAH disease resulted in higher variances due to the weight variation required to calculate SUV. The calculation of SUV refers to radiotracer concentration [MBq / cm^3 ^≙ g] divided by the ratio of injected radioactivity [MBq] to body weight [g]). Due to these aspects, an evaluation by SUV might be unfavorable^26,27^. Since the UR excludes the factors weight and injected radioactivity but only focuses on the enriched radiotracer in general, a data analysis by UR could be more suitable and specific than one by SUV.

The limitation in evaluating image data only after SUV is a common drawback of many image data evaluation programs (e.g., SAMIT—Small Animal Molecular Imaging Toolbox by^28^. Other semi-quantitative parameters, such as the uptake ratio, are usually not calculated by the image evaluation programs and must be calculated manually using additional software. The calculation and output of the UR value in our NU_DPA software can thus be considered a great benefit for the evaluation of [^18^F]FDG accumulation after SAH.

Nowadays, image data are often evaluated in such a way that the image data are aligned on the basis of the additionally acquired anatomical image data due to the higher information content. Such an alignment was not possible here because the acquired image data were solely functional PET image data without anatomical information. Although there are several data analysis programs that allow processing of solely functional PET image data, such as the freely available program AMIDE^29^. AMIDE requires manual alignment of the image data, including alignment of all image data on an atlas. Manual alignment of all image data is not only time-intensive but can also be associated with alignment errors. Furthermore, a determination of the possible volume-of-interest is only possible by geometric bodies (e.g. ellipsoids) and not by anatomical structures. Another alternative for the analysis of the image data could be e.g. the commercially available program PMOD (PMOD Technologies Ltd., Zurich, Switzerland). However, this is associated with high costs.

Due to such limitations, the self-implemented analysis tool NU_DPA was implemented and validated in such a way that it can be used for various medical questions. This includes, among other things, the investigation of cerebral glucose metabolism after experimental subarachnoid hemorrhage over several measurement days and according to various semi-quantitative parameters. This also includes an adequate detection of the VOI (whole brain), as well as the exact subdivision into the individual subregions. By adding and aligning the data on a suitable MR data atlas, it was possible to achieve such suitable evaluation using UR. The alignment of the acquired PET data on a PET data template created of the own PET sham datasets and then aligning them onto the MR atlas using the transformation matrices resulting from the two times affine co-registration process, also increased the validity of the data. Due to the described characteristics of the self-made analysis tool we were able to distinguish metabolic changes in several brain regions. We are aware of the fact that the data analysis was evaluated with a newly developed tool for processing image data. However, the imaging data were again visually verified for correctness and plausibility by two authors independently after each processing step in addition to the output calculation parameters.

We are also aware that normally the PET images with lower spatial resolution are first adapted to the higher spatial resolution of the MR images before co-registration. In contrast, the MRI datasets were resized by a scale factor of 1/20 to match the resolution of the PET image datasets in our analysis. Since the NU_DPA tool is designed for semi-automatic data analysis, matching all PET datasets to the spatial resolution of the high-resolution MR image dataset would not only significantly lengthen the data analysis, but would also not be possible in commercial hardware due to limited random access memory (RAM). Therefore, we decided to register the MRI dataset in the PET template as opposed to the usual procedure. This should be mentioned as a limitation of the data analysis. At the time of the analysis of the PET image data, an alternative use of commercial image data analysis programs, such as PMOD, was not possible for the analysis of the PET image data. This aspect is planned in connection with the further developments of the NU_DPA program.

In addition, this is the first study that shows the quantitative [^18^F]FDG uptake via PET over course of disease up to 7 days following experimental SAH/sham operation. In 1991, Carpenter et al*.* showed by PET in SAH patients on day 2–5 after aneurysm rupture a significant reduction of the cerebral metabolic rate of oxygen (CMRO_2_) of 25% suggesting primary metabolic alterations and an uncoupling of cerebral blood flow (CBF) and metabolism in SAH^30^. To our knowledge, besides the one experimental [^18^F]FDG-PET-study by *Song *et al*.*^25^, there are only three [^18^F]FDG-PET studies performed in SAH patients so far^31–33^. *Enblad *et al*.* investigated simultaneous intracerebral microdialysis (MD) and [^18^F]FDG-PET in the detection of ischemia. In general, they could show that the presence of whole brain ischemia and/or regional ischemia within the region of the MD probe was associated with increased levels of energy-related metabolites (lactate, pyruvate, glucose, adenosine, inosine and hypoxanthine) and excitatory amino acids (EAAs) retrieved by MD ^33^. When PET did not show any signs of ischemia or when signs of regional ischemia were found remote from the MD probe region, only occasionally increased levels of energy-related metabolites and EAAs were seen. They suggested that PET may be of use in defining critical ischemic regions (tissue at risk) where the MD probe could be inserted for chemical monitoring. In 2009 Sarrafzadeh et al. compared the detection of ischemia in 15 aneurysmal SAH patients via MD and ^15^O-H_2_O-PET merged with [^18^F]FDG-PET^32^. They could show that MD parameters were well correlated with glucose hypometabolism and symptoms of ischemia. In 2013 Novak et al*.* used the [^18^F]FDG-PET to elucidate whether aneurysmal SAH-induced vasospasm induces changes of regional glucose uptake in eight surgically clipped patients^31^. They were able to show that SAH-induced vasospasm results in widespread increase of glucose uptake—probably reflecting increased glycolysis. This was detected before neurological focal signs appeared. In severe cases of vasospasm, reflected by neurological deficits, they observed decreased glucose uptake.

In our serial [^18^F]FDG-PET analysis in experimental SAH in rats we could show a persistent increase of [^18^F]FDG uptake up to day 7 in SAH animals, possibly reflecting a hypermetabolism/hyperglycolysis to rebalance the underlying mismatch of energy need and supply. Derangement of metabolism and accumulation of metabolites following SAH have been observed in several studies^30,34–36^. Increased levels of lactate, pyruvate, lactate-pyruvate ratio (LPR) and glutamate in MD studies in SAH patients and experimental SAH in rats have been reported^11,35,36^. In previous studies, we found, that despite incomplete recovery of CBF for more than 6 h after experimental SAH, tissue oxygenation recovered to baseline level 2 h after SAH and significantly exceeded this level up to 140% and more of baseline level after 6 h^37^. The incomplete recovery of CBF which exceeded the decline in tissue oxygenation (ptiO_2_) seems to mirror the disability of O_2_-utilization of the brain. Taken together these findings with our discovery of reduced pyruvate dehydrogenase enzyme (PDH)—key enzyme to the TCA cycle and oxidative phosphorylation—3 h following experimental SAH in rats^12^, we assume that there is a switch from aerobic to anaerobic metabolism. This shift from aerobic to anaerobic metabolism might be due to maintain the energy supply in consideration of the metabolic disturbance starting already in the early phase after SAH (3 h) and lasting at least up to day 7, reflected by the significantly increased [^18^F]FDG uptake in gray matter, neocortex and olfactory system. The fact that the [^18^F]FDG uptake is significantly reduced in white matter and basal forebrain region might be due to the fact, that the ruptured aneurysm, respectively the endovascular punctured vessel to induce SAH, is anatomically located in the frontobasal region and the free ruptured amount of blood flows through the subarachnoid space along the frontobasal olfactory region and further along the outer CSF space reaching gray matter and neocortex. Through diffusion of blood and blood degradation products the white matter is reached, possibly reflected by a reduced [^18^F]FDG uptake. In addition, our findings that aSAH increases the glucose usage in gray matter but decreases it in white matter would be consistent with an impaired function but increased apoptosis—of course with limitations in useful interpretation due to possible underlying anesthetic effects.

Although there might be a bias via the usage of anesthetics such as isoflurane^38^, this experiment would not be possible without anesthesia. The mortality rate of SAH animals would drastically increase without sedation and intubation because of the sharp initial increase of intracranial pressure (ICP) immediately after SAH and the possible consecutive breathing arrest^39^. In addition, PET-scans in SAH rats would not be possible without anesthesia or only with results of enormous blurring. Translational research approaches in SAH patients could try to analyze glucose metabolism via PET scans without anesthetic effects—although not possible in poor grade SAH patients, too.

In a clinical retrospective study on 75 aSAH patients, Wettervik et al. investigated the association between ICP and CPP threshold-insults in relation to cerebral energy metabolism (measured via microdialysis) and clinical outcome after aSAH^40^. They found that higher percent of ICP above 20 mmHg and 25 mmHg thresholds correlated with lower MD-glucose and increased MD-lactate-pyruvate ratio (LPR), particularly between day 4 and day 10 following aSAH. In addition, higher percentage of CPP below 60/70/80/90 mmHg between day 4 to day 10 following aSHA also correlated with a MD pattern of poor cerebral substrate supply and was associated with worse clinical outcome. In a further clinical retrospective study on 60 aSAH patients, Wettervik et al. analyzed the association of the arterial content of oxygen, carbon dioxide, glucose and lactate with cerebral pressure reactivity, energy metabolism and clinical outcome following aSAH^41^. They found that higher pO2 and lower arterial lactate levels were correlated with better cerebral pressure reactivity, but worse energy metabolism. Their results may lead to the conclusion, that agents that promote cerebral vasoconstriction improve an impaired vasodilatory reserve and thereby also pressure reactivity, but vice versa could also lead to a decreased CBF as indicated by the impaired cerebral substrate supply. Undoubtedly it would be interesting to combine experimental setups and analyze if there are any correlations between CBF,CPP, microdialysis and [^18^F]FDG-uptake in PET and future experiments are in preparation.

An important fact of SAH—also in the chosen endovascular filament animal model—is that it leads to a large variability in subarachnoid blood distribution and extent as well as different clinical degrees of severity. Since the focus in our setup was to show metabolic differences in both animal groups for the first time over a period of 7 days, which includes the acute phase of SAH and early brain damage as well as the time points of secondary brain damage and delayed cerebral ischemia over course of disease, which might also be—at least partly—due to metabolic derangement following SAH, we did not add any further measurements/diagnostic tools in our already very complex experimental setup, although this leads to limitations in interpreting our data. Now that we know that the complex interdisciplinary experiment is working, it would be useful to add neurological examinations at the same time points to get a clinical understanding and link to the measured metabolic changes. In addition, it would be an asset to have an additional MRI scan to objectify the amount of subarachnoid blood respectively the severity of SAH and to combine that as well with the neurological performance and clinical severity of SAH, as well with possible onsets of ischemic lesions in the meaning of delayed cerebral ischemia. Otherwise, this would mean an additional possibly stressful examination for the already stressed and injured animal by SAH/Sham operation and early PET scan that might lead to animal loss/drop out and possible bias in the results. Although our number of animals is low and therefor a limitation of the study, we were still able to show several significant results between the two groups (in different brain regions and over several days of disease) that enlarges the power of our study. Nevertheless, additional clinical examination could lead to very inhomogeneous data in such a small group that are difficult to interpret. Therefor further studies should focus on additional imaging (MRI) and neurological performance data in a higher number of animals, too.

With regard to novel neuroprotective therapy options in aSAH, the derangement of cerebral metabolism illustrated in this study could be a potential therapy target. Calcium-channel blockers, like nimodipine, for example, were found to have a beneficial effect in terms of metabolic disruption, histological damage and clinical outcome after cerebral ischemia^42^. While most of the underlying mechanisms are still unknown, also a closer look at the cellular level could be worth it. With regard to our discovery of reduced PDH function, which could play a critical role in the development of an early brain injury following aSAH^12^, the prevention from this inactivity may have a neuroprotective effect. Bypassing the PDH e.g. with dichloroacetate (DCA) or entering “new fuel” to the TCA cycle e.g. via acetyl-L-carnitine (ALCAR) could have neuroprotective effect and attenuate or prevent from secondary brain injury following aSAH^12^. Finally, mitochondrial dysfunction following aSAH is shown to activate the autophagy of neuronal cells, possibly leading to early brain injury and DCI^43^. Therefore, targeting the autophagy-lysosomal system could also have a neuroprotective effect^44^.

## Conclusion

Aneurysmal SAH is a complex cerebrovascular disease with continuing high mortality and morbidity rates. To our best knowledge, this is the first time that metabolic changes following experimental SAH have been examined op to day 7 in time course of disease via [^18^F]FDG-PET and for the first time in different brain regions. We have shown significantly increased [^18^F]FDG uptake in gray matter, neocortex and olfactory system as compared to animals of the sham operated group, while white matter and basal forebrain region showed significantly reduced tracer accumulation in SAH animals. Our PET-template based Nu_DPA program enabled and supported the detection of the changes in glycolysis after aSAH and rebalancing at later time points in five brain regions. Thus, also in view of novel tracer developments, this tool represents a very promising alternative for the evaluation of preclinical data. A better understanding of the underlying metabolic derangement hopefully helps to identify new targets for translational neuroprotective therapies.

## Data Availability

The Matlab scripts of the NU_DPA tool can be requested at the following email (samnick_s@ukw.de).

## Abbreviations

aSAH: Aneurysmal subarachnoid hemorrhage
CBF: Cerebral blood flow
CMRO_2_: Cerebral metabolic rate of oxygen
[^18^F]FDG: 2-[^18^F]Fluoro-2-deoxy-glucose
ICP: Intracranial pressure
LPR: Lactate-pyruvate ratio
MD: Microdialysis
NU_DPA: Nuclear medicine data processing analysis tool
OSEM2D: 2D-ordered subsets expectation maximization
PDH: Pyruvate dehydrogenase enzyme
PET: Positron emission tomography
ptiO_2_: Brain tissue oxygen pressure
SAH: Subarachnoid hemorrhage
SUV: Standardized-uptake-value
TCA: Tricarboxylic acid cycle
UR: Uptake ratio
VOI: Volume-of-interest
